# High-Altitude Extreme Environments Drive Convergent Evolution of Skin Microbiota in Humans and Horses

**DOI:** 10.3390/microorganisms14010057

**Published:** 2025-12-26

**Authors:** Yuwei Zhang, Manyu Zhang, Zhengge Zhao, Yunjuan Peng, Feilong Deng, Hui Jiang, Meimei Zhang, Bo Song, Jae Kyeom Kim, Jeong Hoon Pan, Jianmin Chai, Ying Li

**Affiliations:** 1 Guangdong Provincial Key Laboratory of Animal Molecular Design and Precise Breeding, School of Animal Science and Technology, Foshan University, Foshan 528000, China; 2 Department of Food and Biotechnology, Korea University, Sejong 339770, Republic of Korea; 3 Department of Food and Nutrition, Chosun University, Gwangju 61452, Republic of Korea; jhpan@chosun.ac.kr

**Keywords:** skin microbiota, high altitude, full-length 16S rRNA gene sequencing, Tibetans, Tibetan horses

## Abstract

Unique skin microbial communities have been shaped by the harsh climatic conditions in high-altitude areas, such as intense ultraviolet radiation and low oxygen concentration. However, it is unknown whether high altitude contributes to shaping common microbiota inhabiting the skin across different mammals. The skin microbial communities of humans and horses living in high-altitude (Tibetan) and low-altitude areas were analyzed using full-length 16S rRNA sequencing technology. Alpha diversity differed between high- and low-altitude groups (*p* < 0.01). Skin microbial community composition also differed between high- and low-altitude areas (*p* < 0.05). Some of the common taxa present in the skin of humans and horses in high-altitude areas were identified as extreme microorganisms capable of adapting to the harsh high-altitude environment. Five bacterial taxa, including the genera *Sphingomonas*, *Brevundimonas*, and *Kocuria*, as well as the species *Acinetobacter guillouiae* and *Arboricoccus pini*, were significantly enriched (*p* < 0.01) on the skin of both humans and horses in high-altitude areas. Meanwhile, some taxa enriched on the skin surface at the same altitude showed preferences for mammalian species. *Acinetobacter johnsonii*, *Anaerococcus nagyae*, and *Anaerococcus octavius* were significantly enriched (*p* < 0.05) in the skin of humans at both high and low altitudes, whereas *Acinetobacter pseudolwoffii* and *Armatimonas rosea*, *Archangium gephyra* and *Acinetobacter lwoffii* were significantly enriched (*p* < 0.05) in the skin of horses at both high and low altitudes. In the network analyses, a positive correlation (*p* < 0.01) was shown between the skin taxa enriched in high-altitude areas and each other, while a negative correlation (*p* < 0.01) was found between the skin microorganisms enriched in high-altitude areas and those enriched in low-altitude areas. Overall, our findings indicate that high-altitude extreme environments drive convergent evolution of skin microbiota across mammals, reflecting the joint effects of environmental selection and host-related filtering on community assembly. This cross-species comparison provides a framework for understanding skin microbiome responses to extreme environments in plateau mammals.

## 1. Introduction

Microbial communities represent fundamental drivers of biochemical processes across diverse ecosystems, encompassing human and animal hosts, soil, oceans, and other natural environments [1]. As the primary physical barrier that directly interacts with the external environment in the host [2,3], the skin is colonized by a diverse community of microorganisms, including bacteria, fungi, and archaea [4,5,6]. These microorganisms inhabit distinct biogeographic regions dictated by skin physiological characteristics, and most of them are harmless or even beneficial to the host [7], collectively forming the skin microbiota [8]. This microbiota is of vital importance for maintaining host health [7] as it establishes a symbiotic relationship with the host and regulates multiple physiological functions. For instance, it sustains the skin barrier and reduces water loss by promoting ceramide production—achieved via sphingomyelinase secreted by *Staphylococcus epidermidis* [9]; it participates in immunomodulation by stimulating the immune system and assisting in distinguishing between different types of microorganisms [10]; and it inhibits pathogenic microbes either by competing for nutrient sites or producing antimicrobial substances [11]. The composition of the skin microbiota is influenced by endogenous factors, such as body site and genetics, as well as exogenous factors including lifestyle, diet (a critical nutritional element regulated by nutrigenomics) [12] and climate. Meanwhile, the advancement of sequencing technologies and bioinformatics [13,14] has not only enabled the refined characterization of skin microorganisms [15,16] but also uncovered their complex ecological networks. In particular, emerging sequencing platforms, such as the HiFi-based metagenomic assembly strategy, provide accuracy near isolated genome resolution in metagenome-assembled genomes (MAG) assembly [17], which will greatly facilitate the high-fidelity reconstruction of microbial genomes from complex skin samples and deepen our functional insights into the skin microbiota. Numerous studies have confirmed that the skin microbiota maintains skin microecological balance and overall host health through mechanisms such as immune regulation and skin barrier maintenance [18,19,20].

The extreme environments in high-altitude areas, such as hypoxia, low air pressure, and intense ultraviolet radiation [21,22], can gradually and imperceptibly harm the skin, which serves as the external protective layer of the host. Relevant studies have confirmed that ultraviolet radiation at high altitudes can lead to an increase in the proportion of human skin cancer [23], which fully demonstrates the significant impact of altitude factors on skin health. At the same time, this research has clearly shown that altitude is a key factor affecting the skin microbiota. There are significant differences in the structure and function of the skin microbiota between people (animals) in high-altitude and low-altitude regions [24,25], and the skin microbiota of humans at high altitudes has already demonstrated resistance to ultraviolet damage [26], suggesting that the skin microbiota may play a crucial role in adapting to the extreme conditions of high altitude. Notably, this microbiome-mediated host adaptation to high-altitude habitats has been well validated in yaks—a typical high-altitude mammalian species. A multi-omics study explicitly demonstrated that the microbiome makes a substantial contribution to regulating host metabolic processes, thereby enhancing the species’ tolerance to hypoxic and other extreme high-altitude stresses [27]. The principle that host-associated microbiomes are instrumental in environmental adaptation is further exemplified beyond high-altitude ecosystems: the unique gut microbiome of giant pandas contributes decisively to their dietary adaptation to bamboo through protein metabolism [28]. From high-altitude stress in yaks to specialized nutrition in pandas, these insights firmly support the necessity of exploring whether analogous regulatory mechanisms are orchestrated by the skin microbiota across different high-altitude mammals.

It is worth noting that humans and horses are both mammalian species, and their skin structures and functions are highly similar [29]. As horses are important transportation tools in high-altitude areas and closely associated with human activities, their status as mammals sharing significant similarities with humans in cardiovascular [30], musculoskeletal [31], and skin [25] systems allowed us to jointly validate the effects of altitude on skin microbiota. A previous study has found that horses from high-altitude areas had a higher incidence of skin tumors induced by ultraviolet rays [32], which is similar to the situation where human skin is affected by ultraviolet rays at high altitudes, providing a good foundation for conducting cross-species comparative studies. Furthermore, the skin of horses has abundant sweat glands and sebum secretions, which can provide a complex living environment for microorganisms [32]. In addition, horses possess highly developed sweat glands, and equine sweat is rich in electrolytes such as sodium and chloride [33], which can alter the physicochemical properties of the skin surface and potentially influence skin microbiota colonization and growth. This further enhances its potential as a research subject.

However, the specific differences in the skin microbiome of high- and low-altitude species (including humans and horses) have not yet been fully clarified, and how extreme conditions drive the adaptive changes in the microbial community and the underlying molecular mechanisms remain to be further explored. Therefore, conducting research using the skin microbiome of horses is expected to fill these scientific gaps and systematically reveal the adaptation rules of skin microorganisms in high-altitude environments. To gain a more comprehensive understanding of the impact of altitude on skin microbiota, this study selected humans and horses living at high and low altitudes as research subjects, aiming to explore the microbial adaptation mechanisms under similar high-altitude environmental stress and how these microbiota influence skin health and physiological functions of the host. In terms of technical methods, this study employed more accurate full-length 16S rRNA gene sequencing.

## 2. Materials and Methods

The experiment protocol was approved by the Animal Ethics and Humane Animal Care of the Foshan University (protocol#: FOSU2023693).

### 2.1. Sampling

This study recruited populations of humans and horses from high- and low-altitude areas, which were Daocheng area (high altitude, approximate 3750 masl, latitude 27°58′ N, longitude 99°56′ E) and Guangdong Province (low altitude, approximate 10 masl), China, respectively (Supplementary Tables S1 and S2). All subjects were healthy, without any skin allergies or infections, and those who had used cosmetics within 24 h were excluded. All the participants were given written informed consent. Relevant characteristics, including gender, age, and sampling site, were recorded to exclude individuals with interfering factors in this experiment. In this study, there were a total of 119 human skin samples (high-altitude *n* = 66, low-altitude *n* = 53) and 50 equine skin samples (high-altitude *n* = 21, low-altitude *n* = 29).

The forehead was selected as the exclusive sampling site because it is a highly exposed body region directly subjected to high-altitude stressors, for instance, intense UV radiation and low oxygen. Previous studies have validated that the forehead microbiota exhibits the highest sensitivity to environmental perturbations among multiple body sites, making it an ideal indicator for altitude-driven microbial adaptation [24,34,35,36].

Skin samples were collected by swabbing the forehead of the subjects. First, a sterile cotton swab was soaked in sterile PBS buffer (pH 7.0), and the swab was vigorously rubbed on the sampling site to collect surface substances. The head of the swab after sampling was cut off and placed into a 2 mL centrifuge tube, which was immediately transported in liquid nitrogen. Upon returning to the laboratory, the samples were stored in a −80 °C freezer for further analysis.

### 2.2. Next-Generation Sequencing of 16S Full Length and Bioinformatics

All samples were thawed at 4 °C, and total genomic DNA was extracted based on the instructions of the E.Z.N.A.^®^ soil DNA kit (Omega Bio-tek, Norcross, GA, USA). The concentration and purity of extracted DNA were quantified using a NanoDrop 2000 spectrophotometer (Thermo Fisher Scientific, Waltham, MA, USA). For each sample, a single genomic DNA extraction was conducted to obtain the primary DNA template. After extraction, the quality was assessed by 1% agarose gel electrophoresis, and the concentration and purity were determined using the NanoDrop2000 from Thermo Scientific, USA. Using this DNA as a template, the full-length 16S rRNA gene was amplified by PCR with barcode primers (3 technical replicates per sample). The PCR products were analyzed by 2% agarose gel electrophoresis, purified using magnetic beads, and accurately quantified using Qubit 4.0 (Thermo Fisher Scientific, USA). Subsequently, the samples were mixed in proportion according to the sequencing volume requirements. The mixed samples were used to complete library construction with the SMRTbell Prep Kit 3.0 (Pacific Biosciences, Menlo Park, CA, USA), and sequencing was entrusted to Shanghai Majorbio Bio-pharm Technology Co., Ltd. (Shanghai, China) using the Pacbio Sequel IIe System.

The raw data were analyzed using QIIME 2 (2023.9.1 release) software [31]. First, the raw files were imported into the QIIME 2 platform [37] and saved in QIIME 2’s Artifact format for subsequent analysis. The imported sequences were filtered and dereplicated, and the dereplicated sequences were de novo clustered. Based on a 99% sequence similarity threshold, similar sequences were de novo selected and grouped into operational taxonomic units (OTUs) to generate feature table files and representative sequence files. Then, the uchime-denovo command of the vsearch plugin in QIIME 2 was used, and the UCHIME algorithm [38] was applied to identify and remove chimeric sequences from the clustered sequence data, exporting non-chimeric sequences in fasta format. Through core diversity analysis, alpha and beta diversity indices of microbial communities were calculated. Based on the feature table rarefied to 3600 sequences per sample, ecological differences between samples were evaluated. Target sequences matching the experimental primers were extracted from the 16S reference database Greengenes (gg_24_9) to ensure data comparability, and then a naive Bayesian classifier was trained. OTU sequences were accurately classified through VSEARCH v2.27.1 alignment [38] and the Bayesian model (version 2.12.0) [39]; finally, the classification results were exported for subsequent analysis.

The Shannon index and observed OTUs (operational taxonomic units) were calculated to assess alpha diversity. To correct for differences in sequencing depth, the OTU table was rarefied to a sampling depth of 3600 sequences per sample before calculating alpha diversity indices. To assess beta diversity between samples, distance matrices were constructed based on the Jaccard [40] and Bray–Curtis [41] dissimilarity algorithms, and principal coordinate analysis (PCoA) was employed for visualization.

### 2.3. Statistics

Beta-diversity differences among groups were tested in QIIME 2 using the non-parametric analysis of similarity (ANOSIM), with 999 permutations and statistical significance defined as *p* < 0.05. Differential taxa were identified using LEfSe [42] implemented in the TUTOOL platform [43], with an LDA score > 3.5 and *p* < 0.05 as the significance threshold (Kruskal–Wallis test for overall inter-group comparison and Wilcoxon test for pairwise comparisons). Based on LEfSe outputs, taxa showing notable differences between human and equine groups at high and low altitudes were visualized as boxplots in R (v4.2.0) [43] using ggplot2, and inter-group differences were further assessed using the Wilcoxon rank-sum test (*p* < 0.05). Spearman correlations among taxa were calculated in R (v4.2.0) using psych: corr. test, and correlations were considered significant at *p* < 0.05. Significant correlations were imported into Cytoscape (v3.10.3) [44] to construct association networks, where nodes represent bacterial taxa and edges represent significant correlations.

## 3. Results

### 3.1. Altitude Affects the Diversity of Skin Microbiota of Humans and Horses Living at Different Altitudes

**Figure 1:**
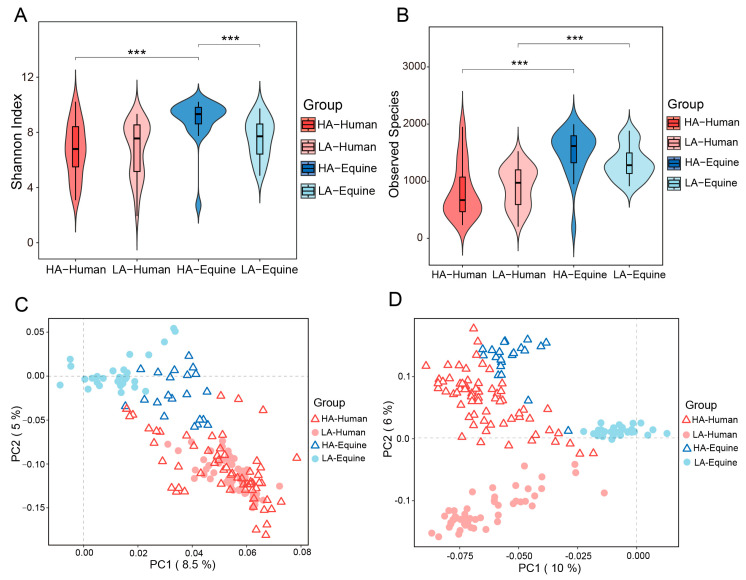
Alpha diversity differences between treatments. Two metrics were used for comparison, including Shannon Index (**A**) and the number of Observed Species (**B**) between high- and low-altitude treatments in both humans and horses. Red and blue bars represent the human and equine skin treatments, respectively. The depth of color represents high and low altitudes, respectively. The principal coordinate analysis (PCoA) plot based on Bray–Curtis (**C**) and Jaccard (**D**) distances shows red and blue colors representing humans and horses, respectively, while circles and triangles indicate high-altitude and low-altitude, respectively. ***: *p* < 0.001, Mann–Whitney U test.

Alpha diversities were calculated to assess the effects among altitude and species. Although there were no significant differences in Shannon index (Figure 1A), observed OTUs (Figure 1B), and Simpson’s index (Supplementary Figure S1) in the skin microbiota between high- and low-altitude humans, both Shannon index and Simpson’s index of skin microbiota of high-altitude horses were significantly higher compared to low-altitude horses (Shannon index *p* < 0.001, Simpson’s index *p* < 0.01). In the meantime, higher diversities of high-altitude equine skin microbial communities were observed compared to high-altitude human skin microbial communities (Shannon index *p* < 0.001, Simpson’s index *p* < 0.01), while low-altitude equine skin microbiota had higher observed OTUs than those of low-altitude humans (*p* < 0.001).

Similarly, significant differences in the structure and membership of skin microbiota was observed between high- and low-altitude treatments for both humans and horses (ANOSIM: Humans: Bray–Curtis R = 0.602, *p* < 0.001; Jaccard R = 0.745, *p* < 0.001; Horses: Bray–Curtis R = 0.941, *p* < 0.001; Jaccard R = 0.952, *p* < 0.001) (Figure 1C,D). Moreover, species effects on beta diversities were also observed, as significant differences between humans and horses living at both high- and low-altitudes were also found (High: Bray–Curtis R = 0.461, *p* < 0.001; Jaccard R = 0.585, *p* < 0.001; Low: Bray–Curtis R = 0.461, *p* < 0.001; Jaccard R = 0.585, *p* < 0.001).

### 3.2. Bacterial Taxonomic Identification Associated with Altitudes and Species

**Figure 2:**
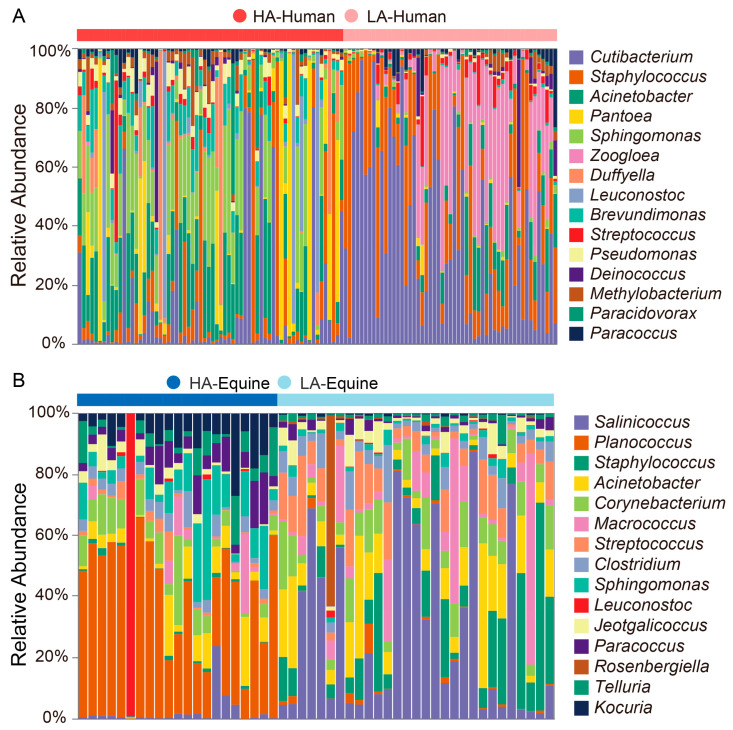
The genus-level microbial composition of samples from humans (**A**) (top 15 genera), with red representing the high-altitude treatment and blue representing the low-altitude treatment; the genus-level microbial composition of samples from horses (**B**) (top 15 genera), with red representing the high-altitude treatment and blue representing the low-altitude treatment.

The bacterial composition was analyzed to identify taxa. At the phylum level, *Bacillota* (humans: 40.3%; horses: 48.5%), *Pseudomonadota* (humans: 34.3%; horses: 22.9%), *Actinomycetota* (humans: 19.0%; horses: 13.1%), and *Bacteroidota* (humans: 1.9%; horses: 4.6%) were the dominant skin phyla in both humans and horses (Supplementary Figure S2). At the genus level, altitude effects contributing to skin microbial composition were observed. For example, the abundances of *Sphingomonas* and *Actinomycetota*, *Planococcus* and *Telluria* were higher in the high-altitude treatment of both humans and horses, while *Cutibacterium* and *Staphylococcus*, *Corynebacterium* and *Streptococcus* were more abundant in the low-altitude treatment (Figure 2). Meanwhile, differences of major taxa between humans and horses were also found. High-altitude humans exhibited significantly higher relative abundances of *Acinetobacter*, *Cutibacterium*, and *Staphylococcus* compared to high-altitude horses, while low-altitude humans had higher relative abundances of *Cutibacterium* and *Zoogloea* than low-altitude horses, and *Thauera* showed a higher abundance in low-altitude horses (Supplementary Figure S3).

### 3.3. Signature Bacteria Differentiating Altitudes and Species

**Figure 3:**
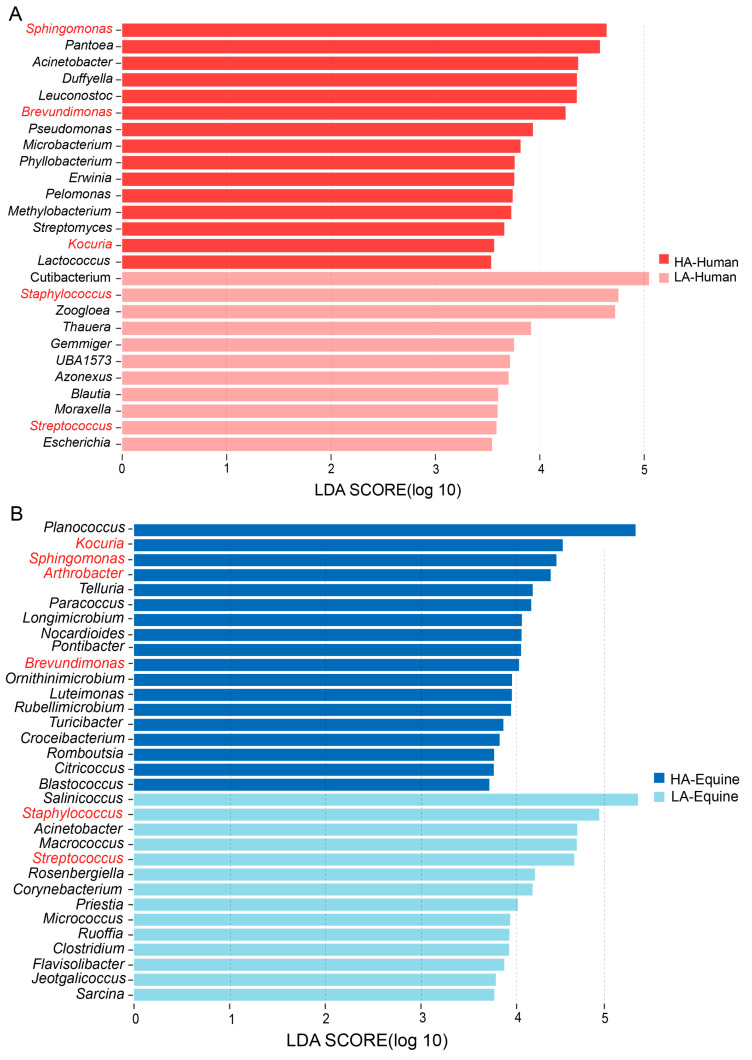
At the genus level, LEfSe analysis (LDA = 3.5) identified microbes with significantly different abundances between the high-altitude (HA) and low-altitude (LA) treatments. Red (**A**) represents human treatments based on altitude, while blue (**B**) represents equine treatments based on altitude. The shade of color indicates the high-altitude treatment and low-altitude treatment, respectively. The red-labeled microbial names in the figure represent species significantly enriched in both humans and horses.

To identify differential microbial taxa between high-altitude and low-altitude treatments, LEfSe analysis was performed on bacterial community data at the genus level (Figure 3). The results showed that 26 genera differed significantly between high- and low-altitude human groups, with 15 genera enriched at high altitude and 11 genera enriched at low altitude. For instance, *Staphylococcus*, *Streptococcus*, and *Cutibacterium* were enriched in the low-altitude treatment. In horses, 32 genera showed significant differences between treatments, with 18 enriched in the high-altitude treatment and 14 enriched in the low-altitude treatment. Interestingly, three genera (*Sphingomonas*, *Brevundimonas* and *Kocuria*) were significantly enriched in both high-altitude humans and horses, while *Staphylococcus* and *Streptococcus* were significantly enriched in low-altitude humans and horses (Supplementary Figure S7), indicating that microbial inhibition was affected by altitude regardless of host species.

Next, to investigate the microbial species differences among treatments, we analyzed the abundances of major microbial species within the differentiating genera identified by LEfSe and combined them with microbial species-level LEfSe output (Supplementary Figure S4). In human skin samples, we found that 7 microbial species were enriched in the high-altitude treatment, such as *Acinetobacter guillouiae*, *Arboricoccus pini* and *Agrobacterium rubi,* while 19 microbial species were enriched in the low-altitude treatment, such as *Acinetobacter johnsonii*, *Acidovorax facilis* and *Anaerococcus octavius*. In equine skin samples, 17 microbial species were enriched in the high-altitude treatment, and 18 microbial species were enriched in the low-altitude treatment. At the same time, shared microbial species at the same altitude were observed regardless of the species differences. For instance, three microbial species (*Acinetobacter guillouiae*, *Arboricoccus pini* and *Agrobacterium rubi*) were detected and abundant in both high-altitude human and equine skin samples.

**Figure 4:**
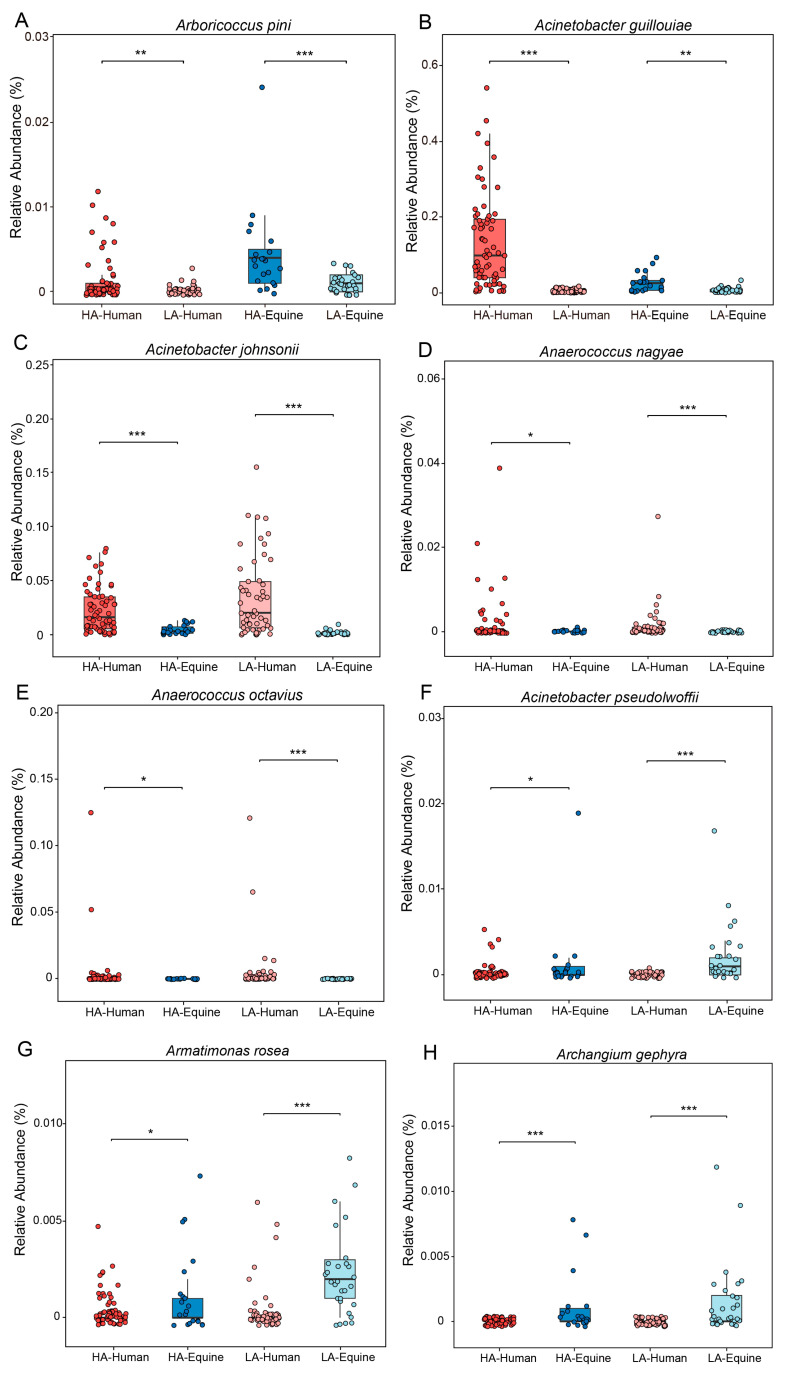
Based on the results of LEfSe analysis (LDA = 3.5), at the species level, *Arboricoccus pini* (**A**), *Acinetobacter guillouiae* (**B**) showed significant differences between high- and low-altitude treatments in both humans and horses. *Acinetobacter johnsonii* (**C**), *Anaerococcus nagyae* (**D**), and *Anaerococcus octavius* (**E**) are bacteria that are significantly and commonly enriched in the skin of people at both high and low altitudes. *Acinetobacter pseudolwoffii* (**F**), *Armatimonas rosea* (**G**), and *Archangium gephyra* (**H**) are bacteria that are significantly and commonly enriched in the skin of horses at both high and low altitudes. *: *p* < 0.05, **: *p* < 0.01, ***: *p* < 0.001; Mann-Whitney U test.

In order to determine the microbial species present on the skin of humans or horses under the same altitude conditions, LEfSe analysis was performed on bacterial community data at the genus level (Supplementary Figure S5). In the high-altitude treatment, we found that 13 genera had higher concentrations in human skin samples, while 22 genera had higher concentrations in equine skin samples, for instance, *Corynebacterium*, *Telluria* and *Macrococcus*. In the low-altitude treatment, 12 genera had higher concentrations in human skin samples, while 17 genera had higher concentrations in equine skin samples. LEfSe analysis was also performed on bacterial community data at the microbial species level (Supplementary Figure S6). Interestingly, two genera (*Cutibacterium* and *Staphylococcus*) were significantly enriched in human skin samples at both high and low altitudes (Supplementary Figure S8), while *Corynebacterium* and *Telluria*, *Macrococcus* and *Salinicoccus*, *Jeotgalicoccus* and *Clostridium*, as well as *Microvirga* were all significantly enriched in equine skin samples regardless of high or low altitude (Supplementary Figure S9), which indicates that specific microorganisms exist in the skin of host species, regardless of the environment. Next, to further investigate the differences in microbial species among different host species, we performed LEfSe analysis at the microbial species level (Supplementary Figure S6). In the high-altitude treatment, we found that 5 microbial species (*Acinetobacter guillouiae*, *Acinetobacter johnsonii*, *Anaerococcus nagyae*, *Anaerococcus octavius* and *Aliterella* sp.) had higher concentrations in human skin samples, while 23 microbial species had higher concentrations in equine skin samples, for instance, *Acinetobacter pseudolwoffii*, *Armatimonas rosea* and *Archangium gephyra*. In the low-altitude treatment, 15 microbial species had higher concentrations in human skin samples, while 18 microbial species had higher concentrations in equine skin samples. Notably, *Acinetobacter johnsonii*, *Anaerococcus nagyae*, and *Anaerococcus octavius* were significantly enriched in the skin of humans at both high and low altitudes (Figure 4). Meanwhile, *Acinetobacter pseudolwoffii, Armatimonas rosea*, *Archangium gephyra* and *Acinetobacter lwoffii* were significantly enriched in the skin of horses at both high and low altitudes (Figure 4, Supplementary Figure S9).

### 3.4. Multiple Taxonomic Treatments Associated with High- and Low-Altitude Samples Show Strong Positive Correlations Respectively

**Figure 5:**
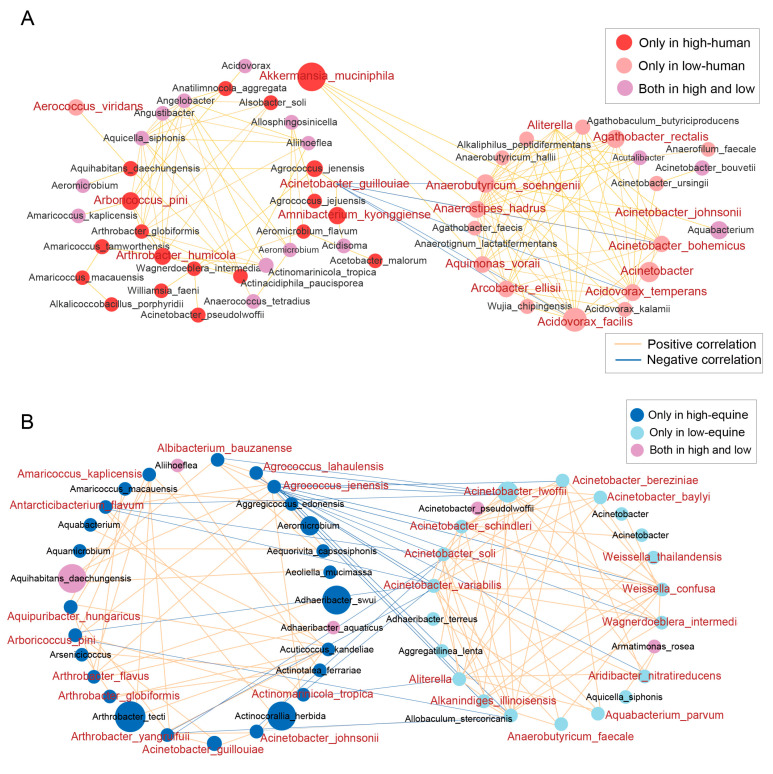
Co-occurrence networks of human (**A**) and equine (**B**) skin microbiota across high and low altitudes were constructed by performing Spearman correlation analysis using bacterial taxa with relative abundances ≥ 0.01 in human and equine skin communities from both altitudes, along with differentially abundant bacteria identified by LEfSe, and visualized via a customized Cytoscape workflow; node size corresponds to microbial relative abundance, and node color indicates ecological distribution: red for bacteria significantly enriched in human skin microbiota (LDA = 2), blue for those enriched in equine skin microbiota, with color intensity representing high or low altitude, and purple for microbial species common to both altitudes; edge width represents the magnitude of Spearman correlation coefficients, where yellow edges denote positive correlations and blue edges denote negative correlations between nodes.

To assess the relationship between the aggregation patterns of microbial communities and altitude, as well as among species, this study employed Spearman correlation analysis. Firstly, it integrated the microbial species with relative abundances of at least 0.01 at two altitudes and those identified by LEfSe. Based on this, a co-occurrence network of human and equine skin microbial communities was constructed, and different correlation thresholds were set. Firstly, altitude effects on the microbial correlations in both humans and horses were estimated, and we found that signature species for high- and low-altitude subjects clustered together with positive correlations (Figure 5). For instance, *Acinetobacter guillouiae*, *Arboricoccus pini*, and *Arthrobacter humicola*, which were significantly enriched in high-altitude human skin microbiomes, clustered together with positive correlations. Conversely, between high and low altitudes, certain microbial species demonstrated negative correlation clustering patterns corresponding to altitude differences. Specifically, in human skin microbiomes, high-altitude *Acinetobacter guillouiae* showed negative correlations with low-altitude microbial species, including *Acidovorax facilis*, *Anaerobutyricum soehngenii*, and *Acinetobacter bohemicus*, forming distinct anti-correlated clusters. These findings indicated that skin microbial communities in high- and low-altitude environments selectively enrich microbial species with environmentally adaptive traits in response to distinct ecological conditions.

Additionally, considering shared microbial species among humans and horses at the same altitudes, we re-constructed Co-occurrence networks to explore the bacterial relationships between species. In species-associated clustering patterns (Supplementary Figure S7), human and equine skin microbiomes at high-altitude each exhibited positive correlation trends, while certain bacterial species showed negative correlations between human and equine skin microbiomes. A similar pattern was observed across species at low-altitude. Moreover, even at the same altitude, skin microbial composition differed significantly between host species.

## 4. Discussion

Numerous studies have demonstrated that altitude can reshape gut microbial communities in humans and other mammals, whereas its influence on skin microbiota remains comparatively underexplored. By jointly analyzing humans and horses inhabiting contrasting elevations, this study demonstrates that altitude-related environmental pressures play a central role in structuring skin bacterial communities, while host species modulate the specific manifestation of these effects. Overall, high-altitude environments were associated with distinct community configurations and the enrichment of specific taxa across hosts, indicating partial convergence driven by shared environmental stressors such as hypoxia and intense ultraviolet (UV) radiation. Differences in alpha diversity reflected this host-dependent modulation rather than a uniform altitude effect: alpha diversity remained stable in humans but increased significantly in horses at high altitude. Together, these findings suggest that altitude acts as a primary ecological filter shaping skin microbiota composition, with host-related factors influencing community diversity patterns and fine-scale taxonomic responses.

Host-specific signatures were observed among taxa that were consistently enriched in humans or horses at a given altitude. For example, *Acinetobacter johnsonii*, *Anaerococcus nagyae* and *Anaerococcus octavius* were repeatedly associated with human skin at both high and low altitudes and have been documented as typical human skin residents [45,46,47]. In the skin microbiota of horses, *Acinetobacter pseudolwoffii* was a bacterium commonly enriched at both high and low altitudes, and relevant literature showed that it can colonize the bodies of domestic animals and has the ability to adapt to the surface and internal environments of domestic animals [48]. Notably, even under the same altitude conditions, the taxa enriched on the skin differed between host species, suggesting that environmental selection alone is insufficient to fully explain skin microbiota assembly. These interspecific differences may be partly driven by host-specific skin physicochemical properties, particularly skin pH. For instance, *Armatimonas rosea* was a bacterium enriched on equine skin, and it was proven that they were suitable for survival in neutral to weakly alkaline environments [49,50], consistent with the skin conditions of horses. Beyond pH, additional host-related factors such as glandular secretions, antimicrobial peptides, immune regulation, and species-specific exposure patterns may further constrain microbial colonization. Together, these host-biased distribution patterns indicate that intrinsic host filtering operates alongside environmental pressures to shape skin microbiota, maintaining stable, species-specific microbial assemblages even under similar high-altitude conditions.

High-altitude environments were associated with convergent enrichment of stress-tolerant bacterial taxa on the skin of both humans and horses. Within high-altitude humans and horses, we identified a set of shared core bacterial taxa that were consistently enriched on the skin of both host species, including *Sphingomonas*, *Brevundimonas*, *Kocuria*, *Acinetobacter guillouiae*, and *Arboricoccus pini*. The recurrence of these taxa across two phylogenetically distinct hosts is consistent with ecological filtering imposed by high-altitude stressors, such as intense UV radiation. Notably, *Acinetobacter guillouiae* was the most frequently detected taxon in both high-altitude humans and horses, indicating a potential ecological advantage under plateau conditions. In support of the relevance of this lineage under high-altitude stress, UV-tolerant *Acinetobacter* strains have been isolated from Andean wetlands located at ~4400 m above sea level, with evidence for antioxidant defense linked to UV tolerance [51]. Previous studies have reported that carotenoid-producing bacteria, such as *Sphingomonas*, *Brevundimonas*, and *Kocuria*, can mitigate UV-associated stress by absorbing harmful radiation, modulating membrane fluidity, and alleviating oxidative damage through scavenging reactive oxygen species [52,53,54,55,56,57]. Consistently, a radiation-resistant *Sphingomonas* species (*Sphingomonas radiodurans*) has been isolated from the north slope of Mount Everest, indicating that members of this genus can persist under high-radiation, high-altitude conditions [58]. In parallel, host-related factors may further shape this microenvironment. Vitamin E [59] and dietary lipids [60] are known to regulate epidermal ceramide synthesis and protect skin lipids from oxidative damage, suggesting that nutritional and microbial mechanisms may act synergistically in skin adaptation [61]. Taken together, our findings and previous evidence suggest that high-altitude conditions are associated with the enrichment of stress-tolerant and carotenoid-producing taxa on the skin. Rather than indicating direct functional adaptation, these patterns likely reflect an ecological filtering process in which both environmental stressors and host skin properties jointly favor microbial taxa with enhanced resilience to UV radiation and oxidative stress.

The diversity patterns observed in our study further emphasize the importance of local environmental context. Although altitude did not markedly change alpha diversity in humans, high-altitude horses displayed richer and more even skin bacterial communities than their low-altitude counterparts. Similar environment-dominated effects on skin microbiota have been reported in amphibians [62], where microbial diversity increases along elevational gradients and appears more strongly driven by habitat than by host species. Interestingly, related studies have shown that the diversity of skin microbiota in high-altitude humans and pigs is significantly lower than that in low-altitude humans and pigs [24,25]. In the study by Zeng et al. [25], the low-altitude human skin samples were collected from Han people in the Sichuan Basin (altitude 319 to 1421 m), while the low-altitude Han human skin samples in our study were from Foshan City, Guangdong Province (altitude 5 to 20 m). Perhaps because Guangdong is located in the South China Sea, at a lower latitude, closer to the equator, and belongs to a subtropical monsoon climate with high annual temperatures, long sunshine hours, and stronger ultraviolet radiation than the Sichuan Basin, the results of this study showed that there was no significant difference in the diversity of skin microbiota between low-altitude and high-altitude humans.

In this study, a more accurate 16S full-length sequencing method was adopted compared with traditional 16S sequencing, which improves taxonomic resolution by covering all variable regions and can precisely distinguish closely related microbial species; it truly reflects the community species abundance by reducing primer bias and lowering PCR amplification preference. Although potential ecological mechanisms underlying altitude-associated microbial patterns are discussed, functional assays were not performed in this study; therefore, these interpretations should be considered ecological inferences rather than direct functional validation. In addition, this study focused on two mammalian hosts, and while cross-species comparisons provide valuable insights, extending the analysis to additional host species will be necessary to assess the generality of altitude-associated skin microbiota patterns.

## 5. Conclusions

Using full-length 16S rRNA gene sequencing, this study demonstrates that skin microbial community composition differs between high- and low-altitude environments and that humans and horses share a subset of taxa enriched under high-altitude conditions. In addition, network analysis reveals distinct co-occurrence patterns among altitude-associated taxa, suggesting structured microbial associations linked to environmental context. Collectively, these findings indicate that high-altitude environmental stress is associated with both shared and host-specific features of skin microbiota in humans and horses, providing a comparative framework for understanding how environmental and host-related factors jointly shape skin microbial communities.

## Data Availability

The raw sequence data reported in this paper have been deposited in the National Center for Biotechnology Information (PRJNA1348721).

## Supplementary Materials

The following supporting information can be downloaded at: https://www.mdpi.com/article/10.3390/microorganisms14010057/s1.
