# Supplementary material for: High-Altitude Extreme Environments Drive Convergent Evolution of Skin Microbiota in Humans and Horses
Source: Microorganisms. 2025 Dec 26;14(1):57. doi: 10.3390/microorganisms14010057 (PMC12843950; doi:10.3390/microorganisms14010057)
Supplement: Supplementary file 1 [file microorganisms-14-00057-s001.zip › microorganisms-4017059-supplementary.pdf]

Supplementary Table S1. The characteristics of human samples

Subject ID	Species	Gender	Age	Sampling site	Altitude
GHB-001-27F-1492	human	male	62	forehead	approximate 3,750 masl
GHB-002-27F-1492	human	male	19	forehead	approximate 3,750 masl
GHB-003-27F-1492	human	male	38	forehead	approximate 3,750 masl
GHB-004-27F-1492	human	male	33	forehead	approximate 3,750 masl
GHB-005-27F-1492	human	female	16	forehead	approximate 3,750 masl
GHB-006-27F-1492	human	female	39	forehead	approximate 3,750 masl
GHB-007-27F-1492	human	female	57	forehead	approximate 3,750 masl
GHB-008-27F-1492	human	male	53	forehead	approximate 3,750 masl
GHB-009-27F-1492	human	female	49	forehead	approximate 3,750 masl
GHB-010-27F-1492	human	male	60	forehead	approximate 3,750 masl
GHB-011-27F-1492	human	female	86	forehead	approximate 3,750 masl
GHB-012-27F-1492	human	female	46	forehead	approximate 3,750 masl
GHB-013-27F-1492	human	female	46	forehead	approximate 3,750 masl
GHB-014-27F-1492	human	female	39	forehead	approximate 3,750 masl
GHB-015-27F-1492	human	female	51	forehead	approximate 3,750 masl
GHB-016-27F-1492	human	female	70	forehead	approximate 3,750 masl
GHB-017-27F-1492	human	male	79	forehead	approximate 3,750 masl
GHB-018-27F-1492	human	male	34	forehead	approximate 3,750 masl
GHB-019-27F-1492	human	female	42	forehead	approximate 3,750 masl
GHB-020-27F-1492	human	male	38	forehead	approximate 3,750 masl
GHB-021-27F-1492	human	female	37	forehead	approximate 3,750 masl
GHB-022-27F-1492	human	female	44	forehead	approximate 3,750 masl
GHB-023-27F-1492	human	male	60	forehead	approximate 3,750 masl
GHB-024-27F-1492	human	male	60	forehead	approximate 3,750 masl
GHB-025-27F-1492	human	male	57	forehead	approximate 3,750 masl
GHB-026-27F-1492	human	female	34	forehead	approximate 3,750 masl
GHB-027-27F-1492	human	female	55	forehead	approximate 3,750 masl
GHB-028-27F-1492	human	female	56	forehead	approximate 3,750 masl
GHB-029-27F-1492	human	female	54	forehead	approximate 3,750 masl
GHB-030-27F-1492	human	female	24	forehead	approximate 3,750 masl
GHB-031-27F-1492	human	female	48	forehead	approximate 3,750 masl
GHB-032-27F-1492	human	female	32	forehead	approximate 3,750 masl
GHB-033-27F-1492	human	female	39	forehead	approximate 3,750 masl
GHB-034-27F-1492	human	female	33	forehead	approximate 3,750 masl
GHB-035-27F-1492	human	female	51	forehead	approximate 3,750 masl
GHB-037-27F-1492	human	male	35	forehead	approximate 3,750 masl
GHB-038-27F-1492	human	female	35	forehead	approximate 3,750 masl
GHB-039-27F-1492	human	female	72	forehead	approximate 3,750 masl
GHB-040-27F-1492	human	female	65	forehead	approximate 3,750 masl
GHB-041-27F-1492	human	male	62	forehead	approximate 3,750 masl
GHB-042-27F-1492	human	female	94	forehead	approximate 3,750 masl
GHB-043-27F-1492	human	male	58	forehead	approximate 3,750 masl

GHB-044-27F-1492	human	male	54	forehead	approximate 3,750 masl
GHB-045-27F-1492	human	female	55	forehead	approximate 3,750 masl
GHB-046-27F-1492	human	female	47	forehead	approximate 3,750 masl
GHB-047-27F-1492	human	male	55	forehead	approximate 3,750 masl
GHB-048-27F-1492	human	female	45	forehead	approximate 3,750 masl
GHB-049-27F-1492	human	female	34	forehead	approximate 3,750 masl
GHB-050-27F-1492	human	female	40	forehead	approximate 3,750 masl
GHB-051-27F-1492	human	female	50	forehead	approximate 3,750 masl
GHB-052-27F-1492	human	male	67	forehead	approximate 3,750 masl
GHB-053-27F-1492	human	female	42	forehead	approximate 3,750 masl
GHB-054-27F-1492	human	male	39	forehead	approximate 3,750 masl
GHB-055-27F-1492	human	male	71	forehead	approximate 3,750 masl
GHB-056-27F-1492	human	female	26	forehead	approximate 3,750 masl
GHB-057-27F-1492	human	female	71	forehead	approximate 3,750 masl
GHB-058-27F-1492	human	female	5	forehead	approximate 3,750 masl
GHB-059-27F-1492	human	female	70	forehead	approximate 3,750 masl
GHB-060-27F-1492	human	male	48	forehead	approximate 3,750 masl
GHB-061-27F-1492	human	female	46	forehead	approximate 3,750 masl
GHB-062-27F-1492	human	female	24	forehead	approximate 3,750 masl
GHB-063-27F-1492	human	female	4	forehead	approximate 3,750 masl
GHB-064-27F-1492	human	male	3	forehead	approximate 3,750 masl
GHB-065-27F-1492	human	male	36	forehead	approximate 3,750 masl
GHB-066-27F-1492	human	female	58	forehead	approximate 3,750 masl
GHB-067-27F-1492	human	male	58	forehead	approximate 3,750 masl
H1-27F-1492	human	female	24	forehead	approximate 10 masl
H3-27F-1492	human	female	25	forehead	approximate 10 masl
H4-27F-1492	human	female	24	forehead	approximate 10 masl
H5-27F-1492	human	female	25	forehead	approximate 10 masl
H6-27F-1492	human	female	23	forehead	approximate 10 masl
H7-27F-1492	human	female	25	forehead	approximate 10 masl
H8-27F-1492	human	female	25	forehead	approximate 10 masl
H9-27F-1492	human	female	24	forehead	approximate 10 masl
H10-27F-1492	human	female	24	forehead	approximate 10 masl
H11-27F-1492	human	female	24	forehead	approximate 10 masl
H12-27F-1492	human	female	26	forehead	approximate 10 masl
H13-27F-1492	human	female	24	forehead	approximate 10 masl
H14-27F-1492	human	female	24	forehead	approximate 10 masl
H16-27F-1492	human	female	27	forehead	approximate 10 masl
H17-27F-1492	human	female	22	forehead	approximate 10 masl
H18-27F-1492	human	female	23	forehead	approximate 10 masl
H19-27F-1492	human	female	23	forehead	approximate 10 masl
H20-27F-1492	human	female	22	forehead	approximate 10 masl
H21-27F-1492	human	female	24	forehead	approximate 10 masl
H22-27F-1492	human	female	22	forehead	approximate 10 masl

H23-27F-1492	human	female	22	forehead	approximate 10 masl
H24-27F-1492	human	female	23	forehead	approximate 10 masl
H25-27F-1492	human	female	25	forehead	approximate 10 masl
H26-27F-1492	human	female	27	forehead	approximate 10 masl
H27-27F-1492	human	female	26	forehead	approximate 10 masl
H28-27F-1492	human	male	27	forehead	approximate 10 masl
H29-27F-1492	human	male	24	forehead	approximate 10 masl
H30-27F-1492	human	male	22	forehead	approximate 10 masl
H31-27F-1492	human	male	25	forehead	approximate 10 masl
H32-27F-1492	human	male	23	forehead	approximate 10 masl
H33-27F-1492	human	male	24	forehead	approximate 10 masl
H34-27F-1492	human	male	25	forehead	approximate 10 masl
H35-27F-1492	human	male	25	forehead	approximate 10 masl
H36-27F-1492	human	male	27	forehead	approximate 10 masl
H37-27F-1492	human	male	24	forehead	approximate 10 masl
H38-27F-1492	human	male	26	forehead	approximate 10 masl
H39-27F-1492	human	male	24	forehead	approximate 10 masl
H40-27F-1492	human	male	25	forehead	approximate 10 masl
H41-27F-1492	human	male	23	forehead	approximate 10 masl
H42-27F-1492	human	male	26	forehead	approximate 10 masl
H43-27F-1492	human	male	27	forehead	approximate 10 masl
H44-27F-1492	human	male	24	forehead	approximate 10 masl
H45-27F-1492	human	male	24	forehead	approximate 10 masl
H46-27F-1492	human	male	24	forehead	approximate 10 masl
H47-27F-1492	human	male	25	forehead	approximate 10 masl
H48-27F-1492	human	male	23	forehead	approximate 10 masl
H49-27F-1492	human	male	26	forehead	approximate 10 masl
M1-27F-1492	human	male	24	forehead	approximate 10 masl
M2-27F-1492	human	male	24	forehead	approximate 10 masl
M3-27F-1492	human	male	26	forehead	approximate 10 masl
M7-27F-1492	human	male	24	forehead	approximate 10 masl
M14-27F-1492	human	male	22	forehead	approximate 10 masl
M25-27F-1492	human	male	25	forehead	approximate 10 masl

Supplementary Table S2. The characteristics of equine samples

Subject ID	Species	Gender	Age	Sampling site	Altitude
GHB-068-27F-1492	horse	male	adult	forehead	approximate 3,750 masl
GHB-069-27F-1492	horse	male	adult	forehead	approximate 3,750 masl
GHB-070-27F-1492	horse	male	adult	forehead	approximate 3,750 masl
GHB-071-27F-1492	horse	male	adult	forehead	approximate 3,750 masl
GHB-072-27F-1492	horse	female	adult	forehead	approximate 3,750 masl
GHB-073-27F-1492	horse	male	adult	forehead	approximate 3,750 masl
GHB-074-27F-1492	horse	male	adult	forehead	approximate 3,750 masl
GHB-075-27F-1492	horse	male	adult	forehead	approximate 3,750 masl
GHB-076-27F-1492	horse	female	adult	forehead	approximate 3,750 masl
GHB-077-27F-1492	horse	male	adult	forehead	approximate 3,750 masl
GHB-078-27F-1492	horse	female	adult	forehead	approximate 3,750 masl
GHB-079-27F-1492	horse	female	adult	forehead	approximate 3,750 masl
GHB-080-27F-1492	horse	female	adult	forehead	approximate 3,750 masl
GHB-081-27F-1492	horse	female	adult	forehead	approximate 3,750 masl
GHB-082-27F-1492	horse	female	adult	forehead	approximate 3,750 masl
GHB-083-27F-1492	horse	female	adult	forehead	approximate 3,750 masl
GHB-084-27F-1492	horse	female	adult	forehead	approximate 3,750 masl
GHB-085-27F-1492	horse	female	adult	forehead	approximate 3,750 masl
GHB-086-27F-1492	horse	female	adult	forehead	approximate 3,750 masl
GHB-087-27F-1492	horse	female	adult	forehead	approximate 3,750 masl

TH-009-27F-1492	horse	female	adult	forehead	approximate 3,750 masl
LR1-27F-1492	horse	male	adult	forehead	approximate 10 masl
LR2-27F-1492	horse	male	adult	forehead	approximate 10 masl
LR3-27F-1492	horse	male	adult	forehead	approximate 10 masl
LR4-27F-1492	horse	male	adult	forehead	approximate 10 masl
LR5-27F-1492	horse	male	adult	forehead	approximate 10 masl
LR6-27F-1492	horse	male	adult	forehead	approximate 10 masl
LR7-27F-1492	horse	male	adult	forehead	approximate 10 masl
LR8-27F-1492	horse	male	adult	forehead	approximate 10 masl
LR9-27F-1492	horse	male	adult	forehead	approximate 10 masl
LR10-27F-1492	horse	male	adult	forehead	approximate 10 masl
LR11-27F-1492	horse	male	adult	forehead	approximate 10 masl
LR12-27F-1492	horse	male	adult	forehead	approximate 10 masl
LR13-27F-1492	horse	male	adult	forehead	approximate 10 masl
LR14-27F-1492	horse	male	adult	forehead	approximate 10 masl
LR15-27F-1492	horse	male	adult	forehead	approximate 10 masl
LR16-27F-1492	horse	male	adult	forehead	approximate 10 masl
LR17-27F-1492	horse	male	adult	forehead	approximate 10 masl
LR18-27F-1492	horse	male	adult	forehead	approximate 10 masl
LR19-27F-1492	horse	male	adult	forehead	approximate 10 masl
LR20-27F-1492	horse	male	adult	forehead	approximate 10 masl
LR21-27F-1492	horse	male	adult	forehead	approximate 10 masl
LR22-27F-1492	horse	female	adult	forehead	approximate 10 masl
LR23-27F-1492	horse	female	adult	forehead	approximate 10 masl
LR24-27F-1492	horse	female	adult	forehead	approximate 10 masl
LR25-27F-1492	horse	female	adult	forehead	approximate 10 masl
LR26-27F-1492	horse	female	adult	forehead	approximate 10 masl
LR27-27F-1492	horse	male	adult	forehead	approximate 10 masl
LR28-27F-1492	horse	male	adult	forehead	approximate 10 masl
LR29-27F-1492	horse	male	adult	forehead	approximate 10 masl

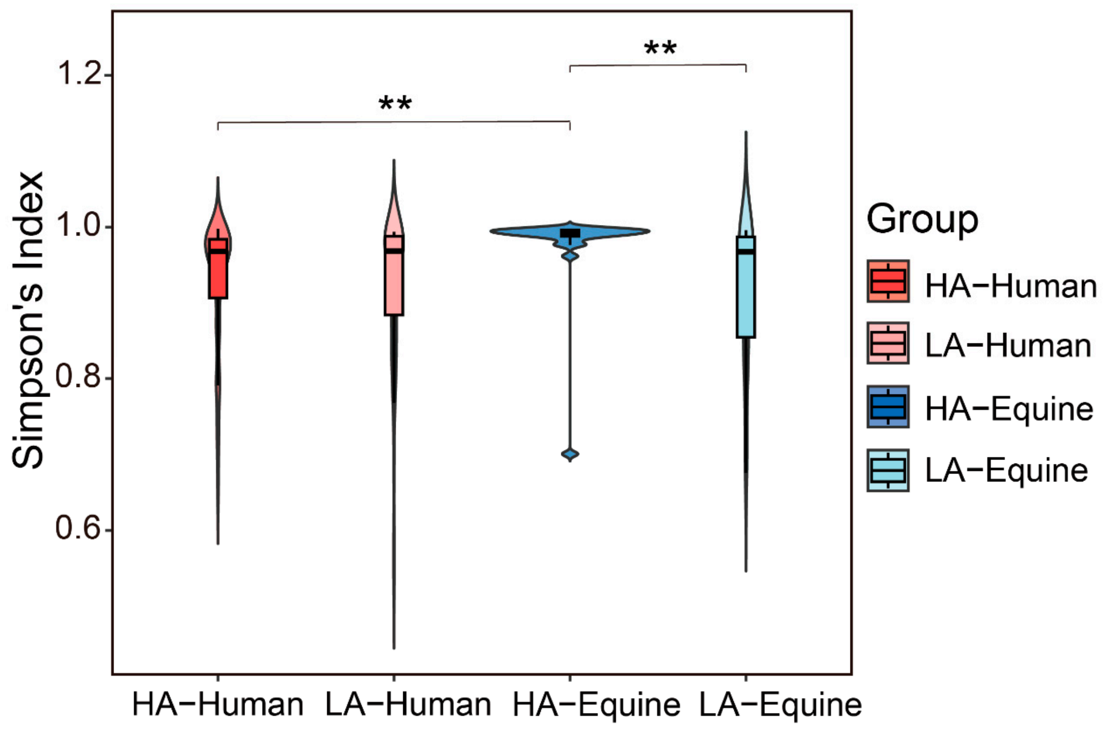

Supplementary Figure S1. Alpha diversity differences between treatments were analyzed and compared using the Simpson index (A).
*:$p < 0.05$, **:$p < 0.01$, ***:$p < 0.001$, Mann-Whitney U test

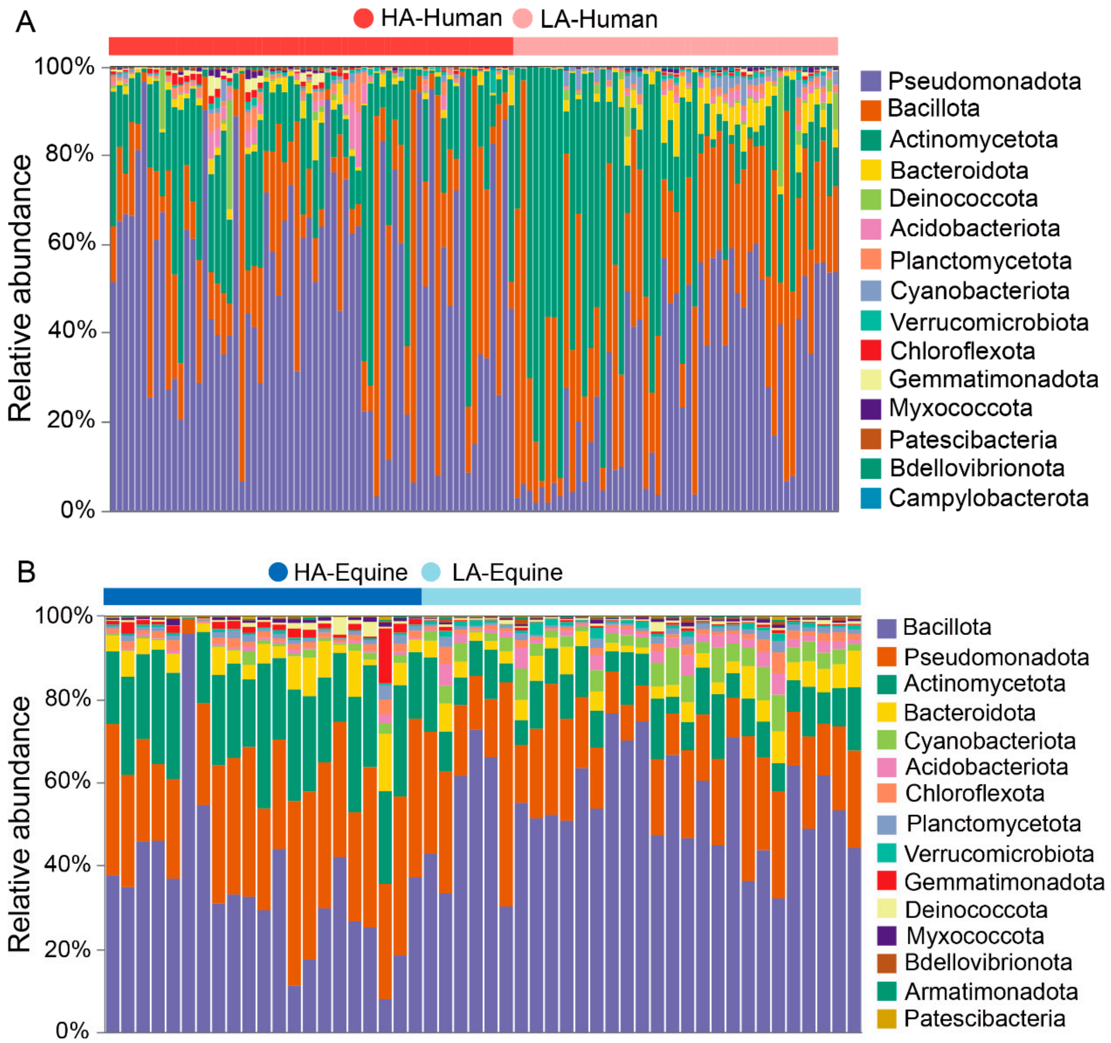

Supplementary Figure S2. The phylum-level microbial composition of samples from humans (A) (top 15 taxa), the phylum-level microbial composition of samples from horses (B) (top 15 taxa), In the figure, colors distinguish the human skin treatment (red) and the equine skin treatment (blue). The depth of the color represents high altitude and low altitude, respectively.

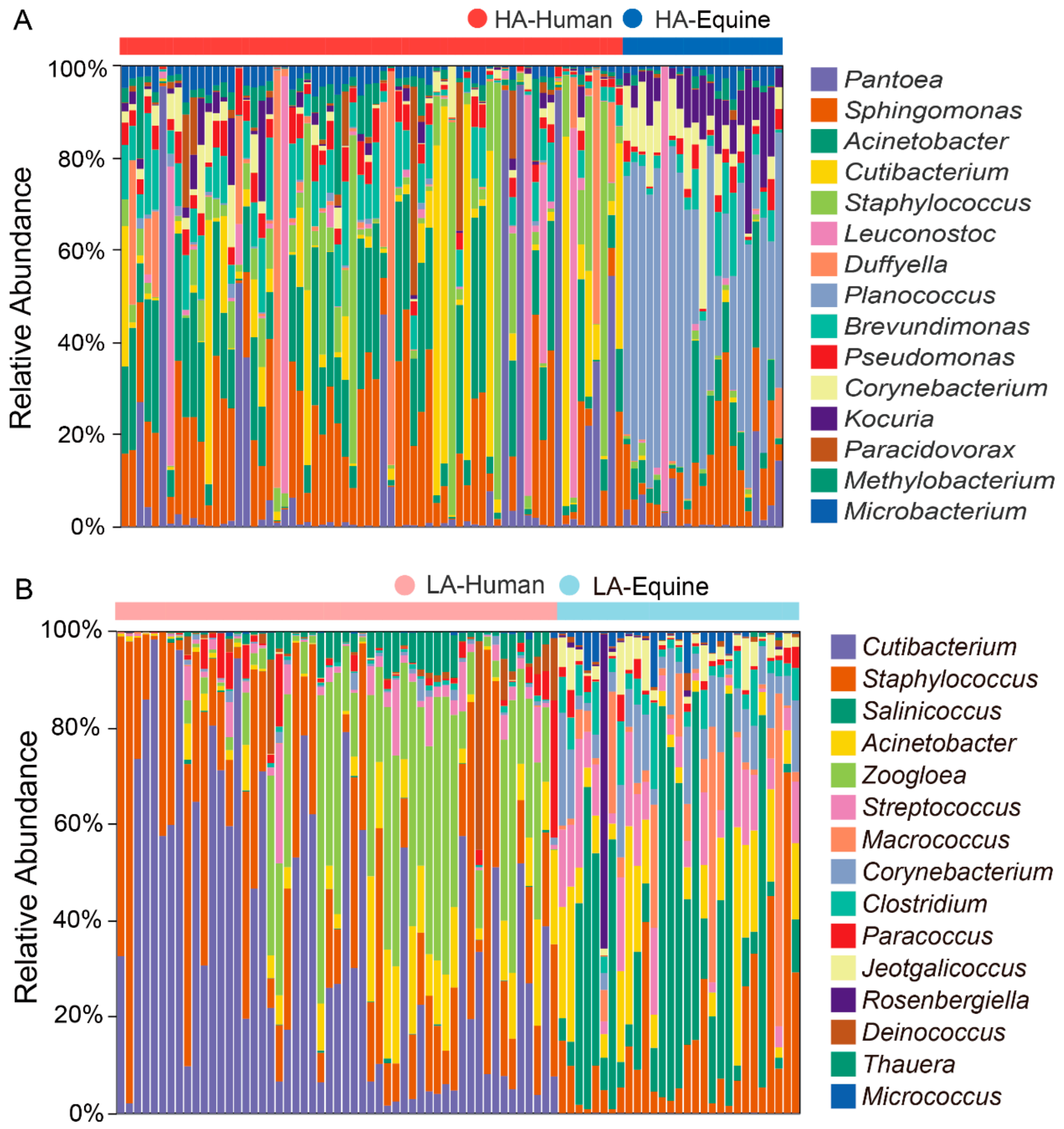

Supplementary Figure S3. The genus-level microbial composition of samples from humans (A) (top 15 taxa), the genus-level microbial composition of samples from horses (B) (top 15 taxa), In the figure, colors distinguish the human skin treatment (red) and the equine skin treatment (blue). The depth of the color represents high altitude and low altitude, respectively.

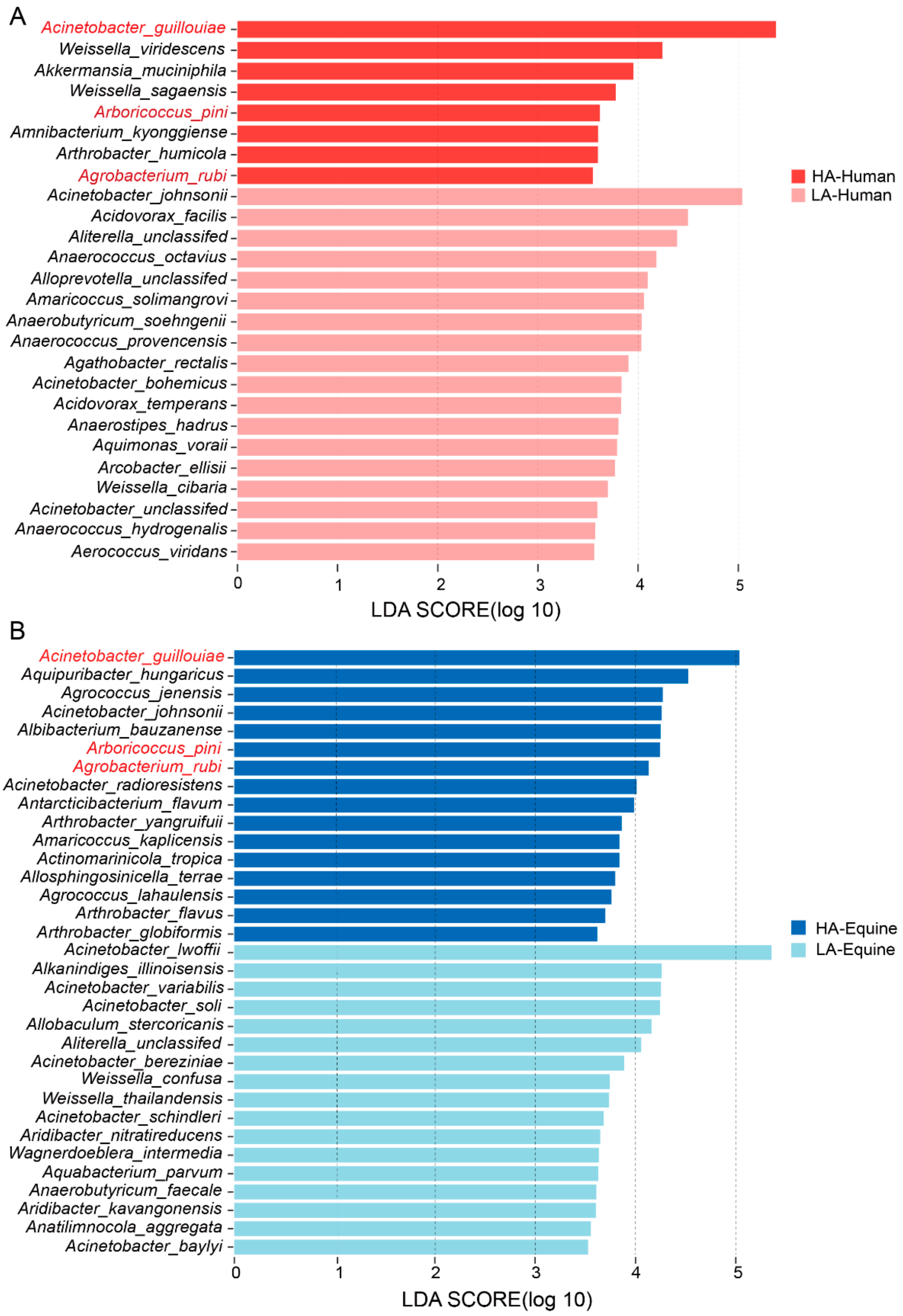

Supplementary Figure S4. At the altitude level, LEfSe analysis (LDA value = 3.5) identified microbes with significantly different abundances between the high-altitude (HA) and low-altitude (LA) treatments. Red (A) represents human treatments based on altitude, while blue (B) represents equine treatments based on altitude. The shade of color indicates the high-altitude treatment and low-altitude treatment, respectively. The microorganisms marked in red in the figure represent the bacteria that are commonly enriched in the skin of both humans and horses at high altitudes.

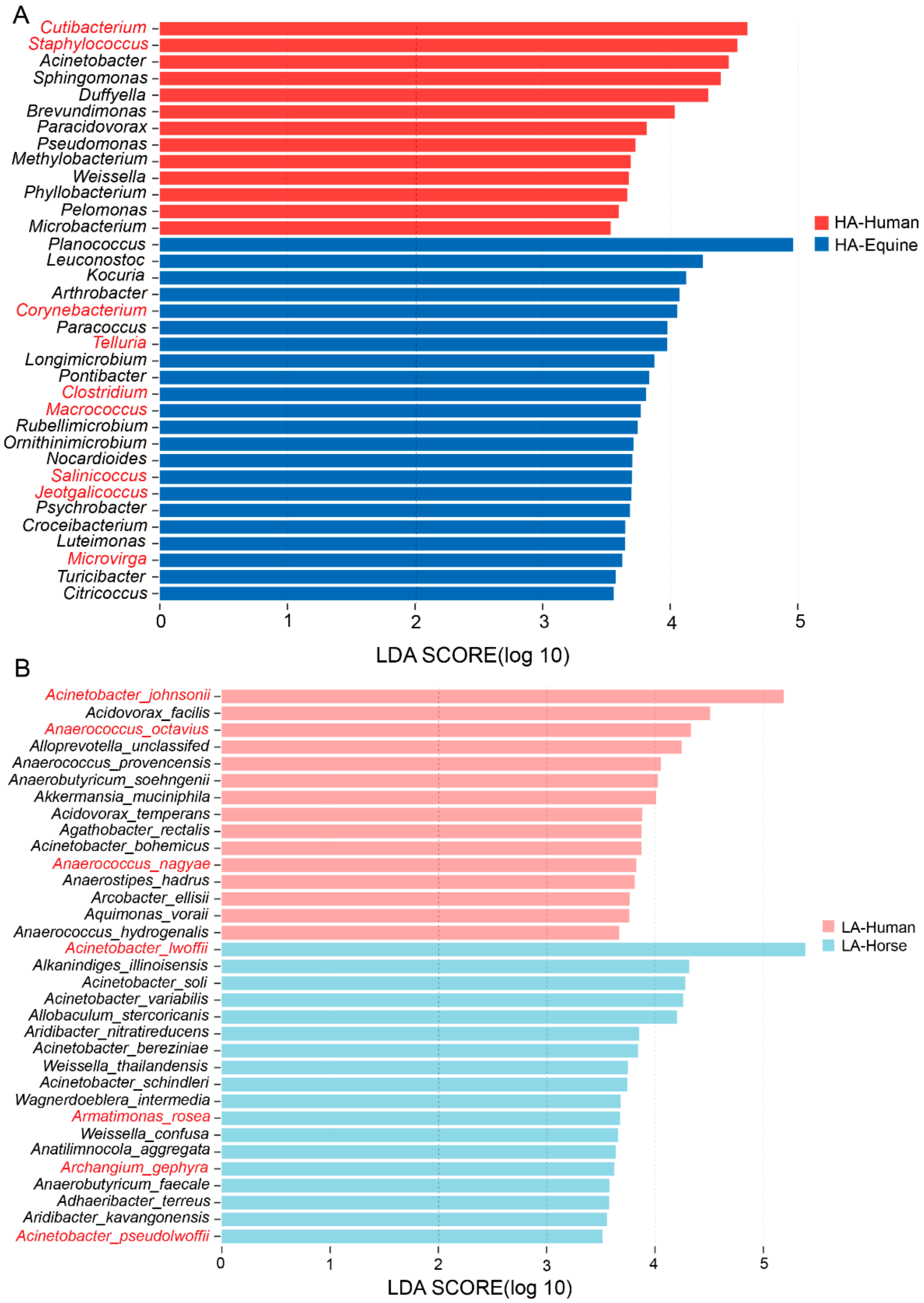

Supplementary Figure S5. At the genus level, the LEfSe analysis (with an LDA value of 3.5) revealed significant differences in the skin microbiomes of humans and horses. The darker part (A) represents the treatment based on high-altitude humans and horses, while the lighter part (B) represents the treatment based on low-altitude humans and horses. Red and blue represent the treatment groups for humans and horses respectively. The names of the microorganisms with red labels in the figure represent the bacteria that are commonly enriched in both humans at high and low altitudes, as well as in horses at high and low altitudes.

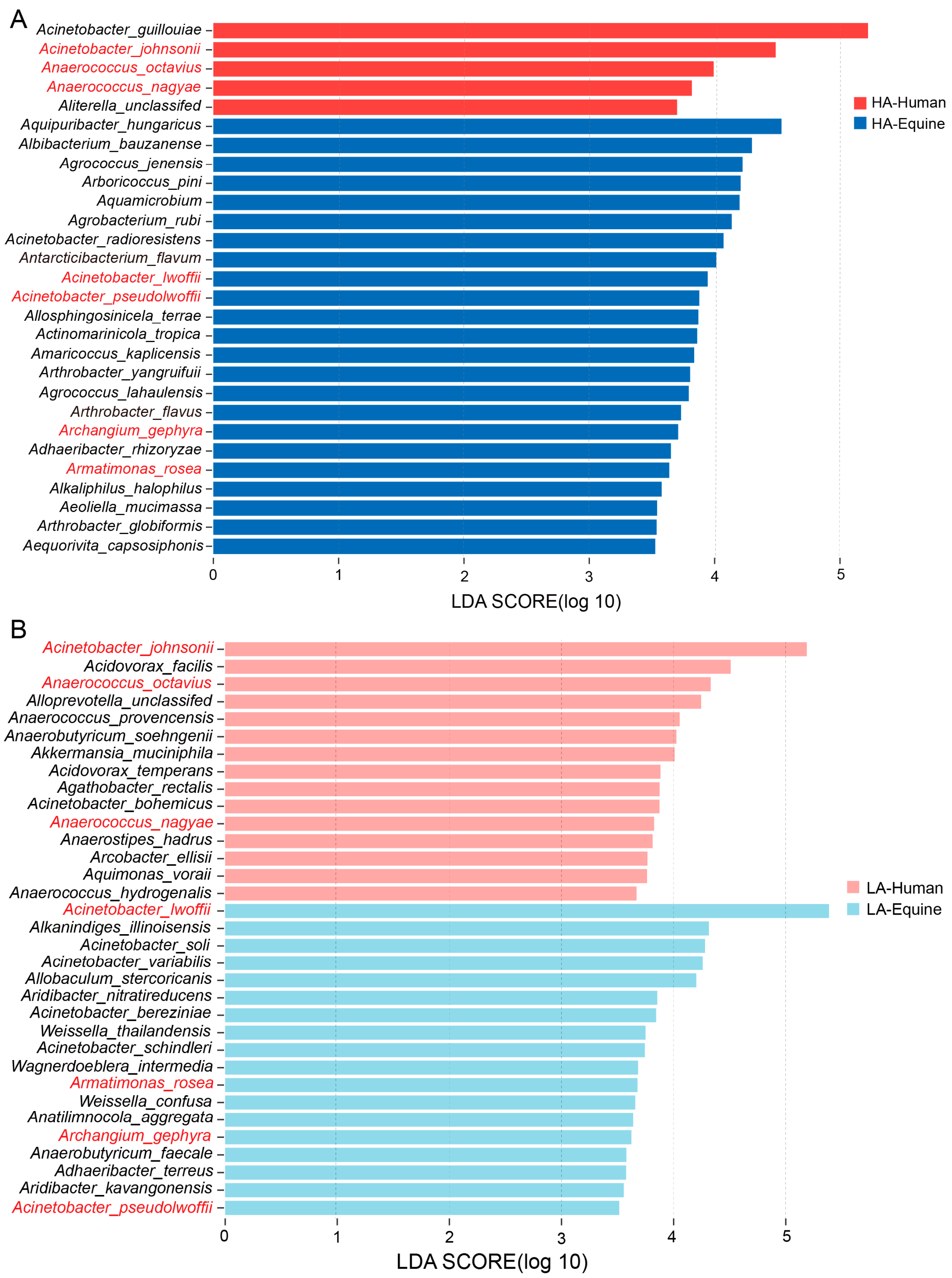

Supplementary Figure S6. At the species level, the LEfSe analysis (with an LDA value of 3.5) revealed significant differences in the skin microbiota between humans and horses. The dark (A) represents the treatment based on high-altitude humans and horses, while the light (B) represents the treatment based on low-altitude humans and horses. Red and blue represent the treatment groups for humans and horses respectively. The names of the microorganisms with red labels in the figure represent the bacteria that are commonly enriched in both humans at high and low altitudes, as well as in horses at high and low altitudes.

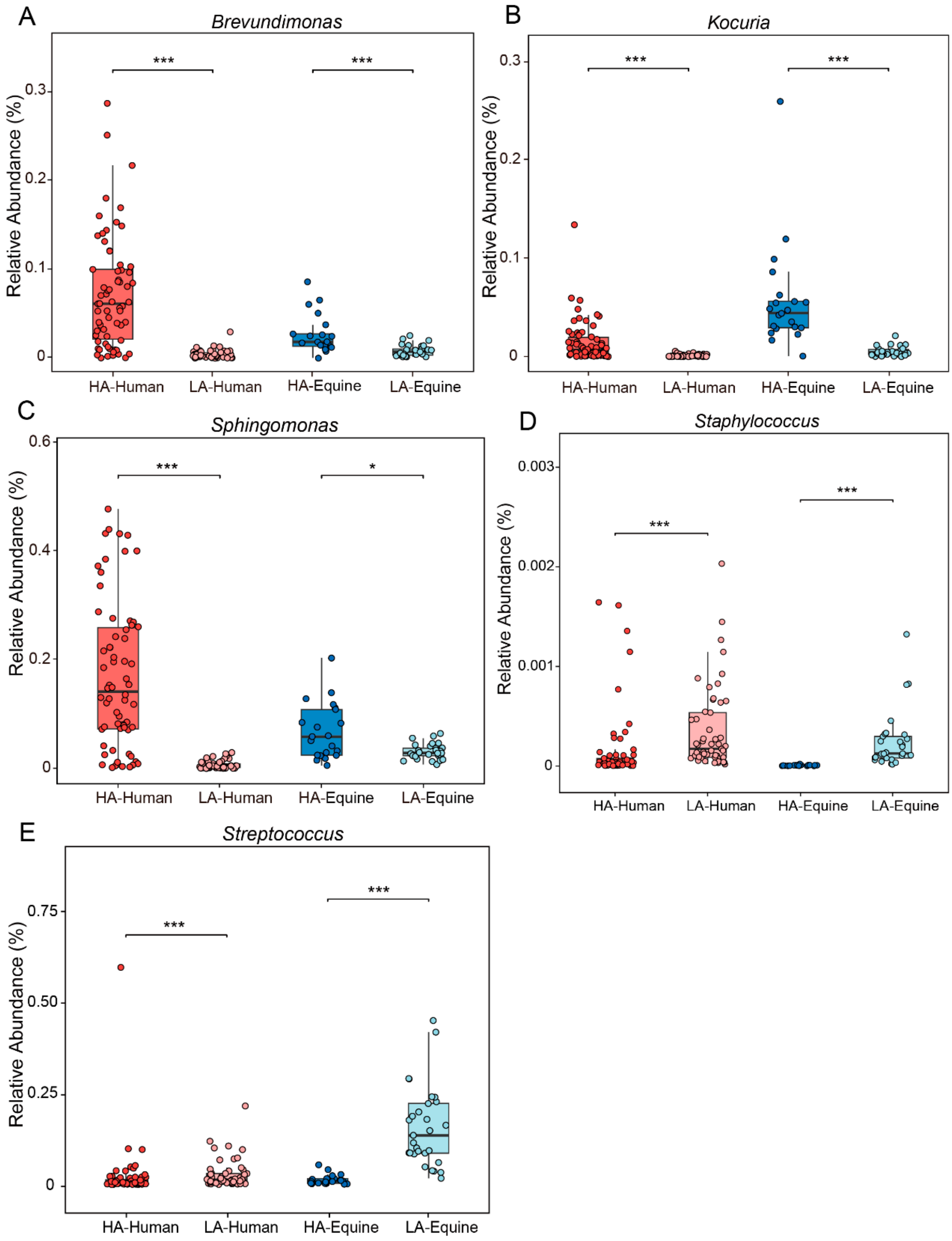

Supplementary Figure S7. Based on the results of LEfSe analysis (LDA = 3.5), at the genus level, *Brevundimonas* (A), *Kocuria* (B), and *Sphingomonas* (C) were significantly enriched in the skin of humans and horses at high altitudes, while Staphylococcus and Streptococcus were significantly enriched in the skin of humans and horses at low altitudes.

*:$p < 0.05$, **:$p < 0.01$, ***:$p < 0.001$, Mann-Whitney U test

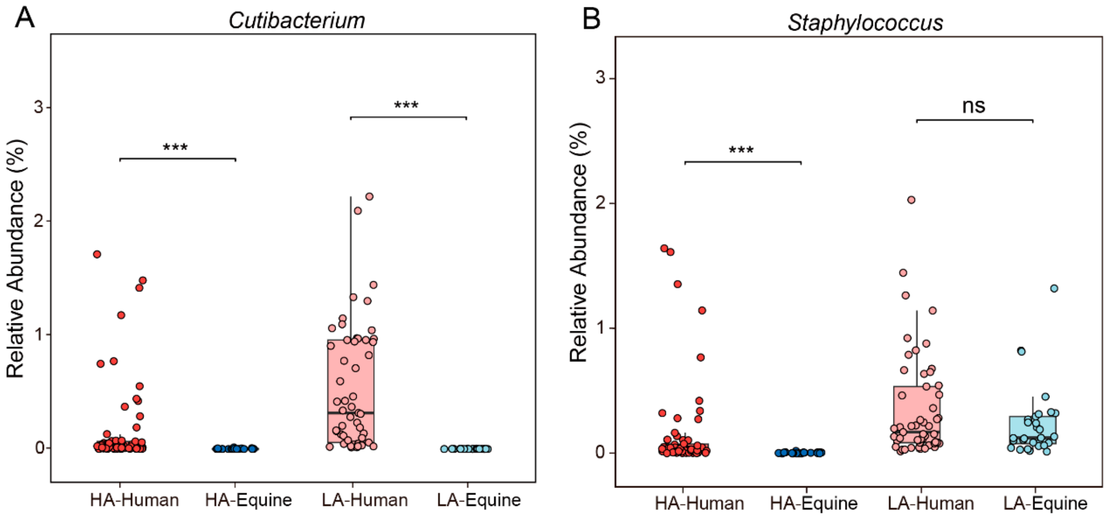

Supplementary Figure S8. Based on the results of LEfSe analysis (LDA = 3.5), at the genus level, Cutibacterium (A) and Staphylococcus (B) were significantly enriched in both high-altitude and low-altitude human skin.

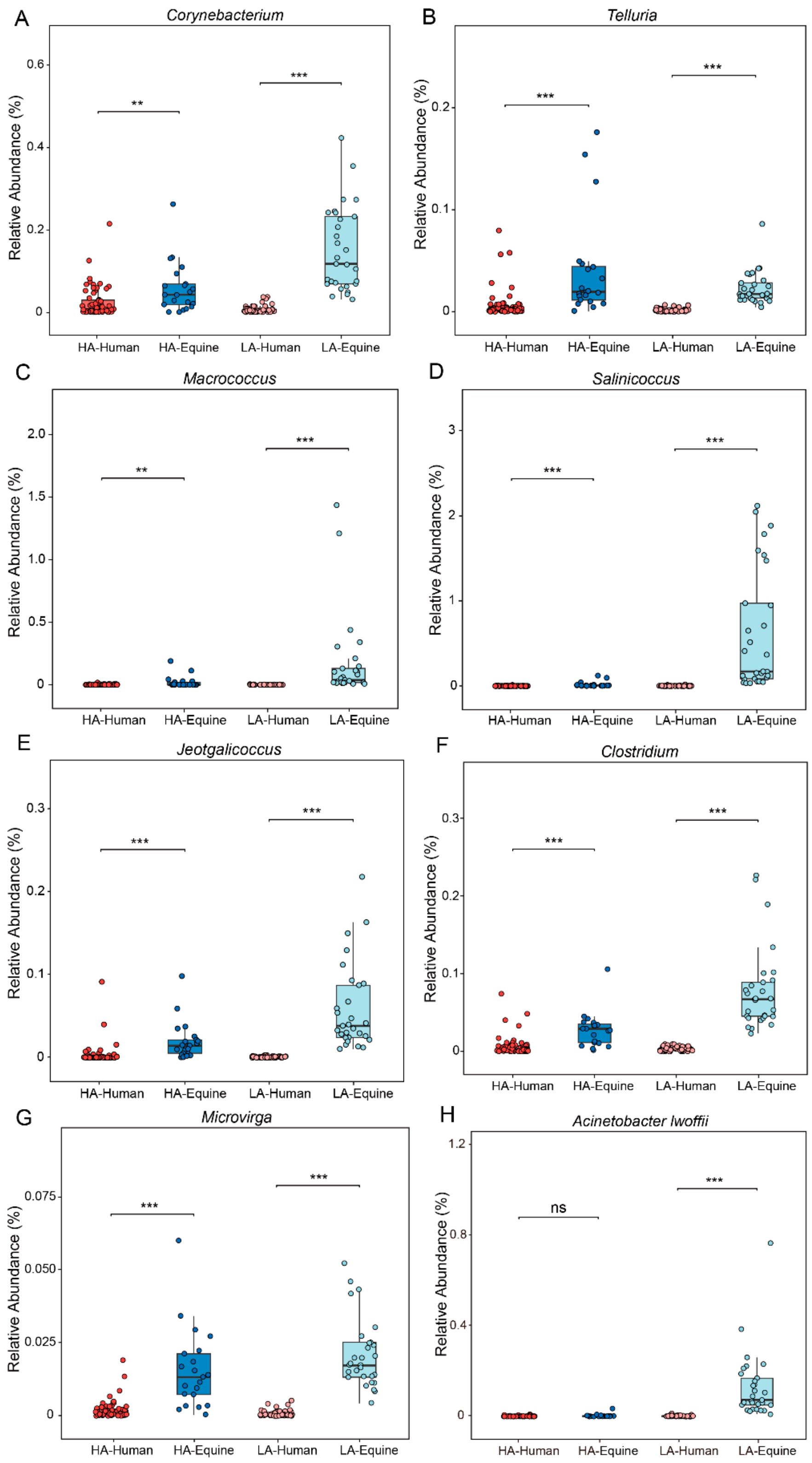

Supplementary Figure S9. Based on the results of LEfSe analysis (LDA = 3.5), at the genus level, Corynebacterium (A) and Telluria (B), Macrococcus (C) and Salinicoccus (D), Jeotgalicoccus (E) and Clostridium (F), Microvirga (G) were significantly enriched in both high-altitude and low-altitude equine skin. At the genus level, Acinetobacter lwoffii (H) was significantly enriched in both high-altitude and low-altitude equine skin.

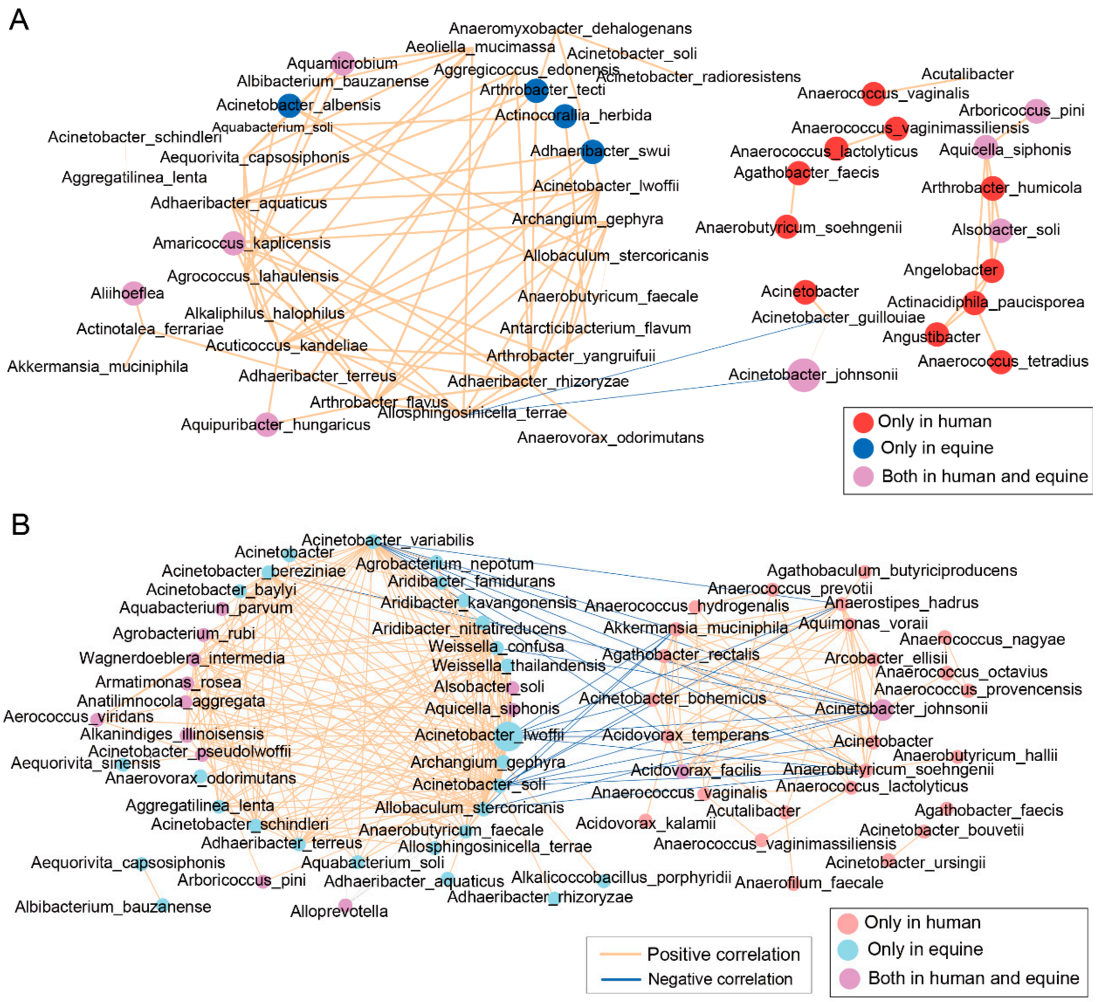

Supplementary Figure S10. Co-occurrence networks of human and equine skin microbiota across high and low altitudes were constructed by performing Spearman correlation analysis (human: |rho| > 0.5, horse: |rho| > 0.5) using bacterial taxa with relative abundances ≥ 0.01 in human and equine skin communities from both altitudes along with differentially abundant bacteria identified by LEfSe, and visualized via a customized Cytoscape workflow; node size corresponds to microbial relative abundance, and node color indicates ecological distribution: red for bacteria significantly enriched in human skin microbiota (*LDA = 2*), blue for those enriched in equine skin microbiota, with color intensity representing high or low altitude, and purple for taxa common to both altitudes; edge width represents the magnitude of Spearman correlation coefficients, where yellow edges denote positive correlations and gray edges negative correlations between nodes.
